# Hypoglycemic, Antioxidant Activities, and Probiotic Characteristics of *Lacticaseibacillus rhamnosus* LBUX2302 Isolated from Stool Samples of Neonates

**DOI:** 10.3390/life15050804

**Published:** 2025-05-18

**Authors:** Pedro A. Reyes-Castillo, Ana Laura Esquivel-Campos, Edgar Torres-Maravilla, Eduardo Zúñiga-León, Felipe Mendoza-Pérez, Rosa González-Vázquez, María Guadalupe Córdova-Espinoza, María Angélica Gutiérrez-Nava, Raquel González-Vázquez, Lino Mayorga-Reyes

**Affiliations:** 1 Doctorado en Ciencias Biologicas y de la Salud, Universidad Autonoma Metropolitana, Mexico City 04960, Mexico; 2202802526@alumnos.xoc.uam.mx; 2 Laboratorio de Biotecnologia, Departamento de Sistemas Biologicos, Universidad Autonoma Metropolitana Unidad Xochimilco, Mexico City 04960, Mexico; aesquivel@correo.xoc.uam.mx (A.L.E.-C.); jezuniga@correo.xoc.uam.mx (E.Z.-L.); fmendoza@correo.xoc.uam.mx (F.M.-P.); 3 Facultad de Medicina Mexicali, Universidad Autonoma de Baja California, Mexicali 21000, Mexico; edgar.torres.maravilla@uabc.edu.mx; 4 Laboratorio de Bacteriologia Medica, Escuela Nacional de Ciencias Biologicas, Instituto Politecnico Nacional (IPN), Mexico City 11350, Mexico; rosagonzvazq@yahoo.com.mx (R.G.-V.); mixtlipp@yahoo.com.mx (M.G.C.-E.); 5 Unidad Medica de Alta Especialidad, Hospital de Especialidades, “Dr. Antonio Fraga Mouret”, Centro Medico Nacional La Raza, Instituto Mexicano del Seguro Social (IMSS), Mexico City 02990, Mexico; 6 Laboratorio de Inmunologia, Escuela Militar de Graduados de Sanidad, Mexico City 11200, Mexico; 7 Laboratorio de Ecologia Microbiana, Departamento de Sistemas Biologicos, Universidad Autonoma Metropolitana Unidad Xochimilco, Ciudad de Mexico 04960, Mexico; agutz@correo.xoc.uam.mx; 8 Laboratorio de Biotecnologia, Departamento de Sistemas Biologicos, Secihti-Universidad Autonoma Metropolitana Unidad Xochimilco, Mexico City 04960, Mexico

**Keywords:** *Lacticaseibacillus rhamnosus*, probiotics, bile salt hydrolase activity, antioxidant effect, adhesion

## Abstract

*Lacticaseibacillus* species have shown potential in managing hyperglycemia, hypercholesterolemia, and oxidative stress, depending on the strain and species. This study aimed to isolate a novel *Lacticaseibacillus rhamnosus* strain from healthy newborns and assess its hypoglycemic and antioxidative activity, along with other probiotic properties. A non-hemolytic *L. rhamnosus* LBUX2302 was isolated, and it exhibited survival rates of 2.7%, 22%, and 27.5% at pH 2, 3, and 5 for 120 min. It metabolized various carbon sources and showed resistance to gentamicin, dicloxacillin, and penicillin; coaggregated with *Salmonella typhi* ATCC14028, *Staphylococcus aureus* STCC6538, and *Escherichia coli* O157:H7. *L. rhamnosus* LBUX2302 showed hydrophobicity, autoaggregation, and adhesion to HaCat, HeLa, MCF-7, SK-LU-1, and SW620 cell lines. It also exhibited extracellular activity of bile salt hydrolase. Enzymatic inhibition assays revealed 66% and 24% inhibitions of α-amylase and α-glucosidase, respectively. Its cell-free supernatant inhibited DPPH (89%), hydroxyl (81%), and superoxide anion radicals (61%). Also, antioxidant activity was observed in whole cells and cell fragments. Finally, the presence of ferulic acid activity was detected. The results highlight *L. rhamnosus* LBUX2302 as a promising probiotic with hypoglycemic and antioxidant effects, warranting further in vivo evaluation for its possible inclusion in functional food and health formulations.

## 1. Introduction

Lactic acid bacteria (LAB) comprise the *Lacticaseibacillus* genus, in which the *rhamnosus* species is included [1]. LAB have been isolated from various sources, such as fermented dairy and non-dairy products, the gastrointestinal tract of humans, animals, and insects, as well as human breast milk [2,3]. FAO/WHO suggested that potential probiotics should be capable of surviving passage through the digestive tract. This means they must be resistant to gastric juices and be able to grow in the presence of bile under conditions found in the intestines. As with any bacteria, antibiotic resistance exists among some LAB, and this resistance may be related to chromosomal- or plasmid-located genes [4].

Subsequently, their functional properties should be assessed, including adhesion to the epithelial surface, antimicrobial and antioxidant activity, hydrophobicity, and self-aggregation [5,6]. LAB can attach to the intestinal epithelium via various mechanisms, including passive forces, electrostatic and hydrophobic interactions, steric forces, lipoteichoic acids, and distinct surface structures. This ability to adhere may inhibit pathogenic bacteria from binding to intestinal cells [7].

LAB can reduce cholesterol through the enzymatic activity of bile salt hydrolase (BSH) [8]. Certain *Lacticaseibacillus* strains have shown hypoglycemic potential, attributed to their ability to inhibit the enzymatic activity of α-amylase and α-glucosidase. This inhibition slows glucose uptake, promoting a more stable glycemic profile, and suggests a potential therapeutic application for the management of type 2 diabetes mellitus (T2DM) [9,10]. T2DM and oxidative stress are closely linked due to hyperglycemia-induced overproduction of reactive oxygen species (ROS), such as superoxide anion (O^−2^), hydrogen peroxide (H_2_O_2_), and hydroxyl radicals (OH^−^). Some *Lacticaseibacillus* strains have shown antioxidant activity by increasing different free radical scavenging activities and superoxide dismutase enzyme activity, which could help to reduce oxidative stress, thus impacting glucose homeostasis [11].

*L. rhamnosus* isolated from cheese showed antibacterial effects against *Escherichia coli, Staphylococcus aureus*, and *Pseudomonas aeruginosa* [12]. *Lacticaseibacillus rhamnosus LR22* has been reported as beneficial against constipation [13]. Due to the above, this work focused on isolating a new strain of *L. rhamnosus* from healthy newborns and evaluating its hypoglycemic and antioxidative activity, along with other probiotic properties.

## 2. Materials and Methods

### 2.1. Strain Isolation

LAB isolation was performed using stool samples from healthy newborns from an obstetric clinic of the public health service in Mexico. The research protocol was approved by the Ethics Committee of the clinic under registration number 36,068. Details about isolation are found in Reyes-Castillo et al. (2023) [10]. We did not handle the stool samples directly as we received the purified strains, which were subsequently examined for their morphology and Gram staining characteristics.

### 2.2. Molecular Identification

A Wizard^®^ Genomic DNA Purification Kit (Promega, Tokyo, Japan) was used to perform gDNA extraction, following the manufacturer’s protocol. The primers used to amplify a segment of the genomic DNA of our isolated strain were 27F (AGAGTTTGATCMGGCTCAG) and 1491R (TACGGYTACCTTGTTAGGATT). The conditions for the PCR reaction were according to Galkiewicz and Kellogg, 2008 [14]. The amplified product was sequenced by the Integrated Microbiome Resource (Halifax, NS, Canada). Sequence alignment and comparisons were conducted using MEGA5 and NCBI’s Basic Local Alignment Search Tool (http://blast.ncbi.nlm.nih.gov/Blast.cgi) (accessed on 18 May 2024).

### 2.3. Phylogeny Analysis

The phylogenetic tree was constructed using MEGA software (version 11.0.13) [15] by the Maximum Likelihood method with the Kimura 2-parameter model [16]. The 16S rRNA gene sequences were obtained from seven *Lactobacillus* species deposited in the NCBI database. Bootstrap values of the tree were computed by resampling 1000 replications.

### 2.4. Catalase Test

A 12 h culture plate of pure bacterial cells was used to evaluate catalase activity by placing a drop of hydrogen peroxide on the center of a clean slide. Then, using a sterile bacteriological loop or stick, a small amount of the bacterial colony was transferred and mixed with the H_2_O_2_ drop. The reaction was observed immediately. The positive control was *Staphylococcus aureus* ATCC6538 [17], while *Lacticaseibacillus casei* isolated from a commercial productwas the negative control. This strain was used as the control in the next experiments. Catalase-positive bacteria produced bubbles due to the release of oxygen, whereas catalase-negative bacteria did not generate bubbles. Triplicates of the test were performed [18].

### 2.5. Hemolysis Test

A bacterial suspension containing 1 × 10^9^ CFU/mL was seeded onto blood agar and incubated at 37 °C for 48 h. *S. aureus* ATCC6538 [17] and *L. casei* [18] were used as controls. Hemolysis was considered positive when complete red blood cell lysis was observed, whereas the absence of hemolysis was classified as a negative result. Triplicates of the test were performed [10].

### 2.6. Growth Kinetics on Different Carbon Sources

Bacterial growth was monitored over 10 h at 2 h intervals using glucose, sucrose, fructooligosaccharides (FOS), lactose, lactulose, and raffinose at 0.01% (*w*/*v*) concentrations in a minimal medium (pH 7) containing 0.02 g/L NaCl, 1 g/L (NH_4_)_2_ SO_4_, 0.02 g/L, CaCl_2_ ۔ H_2_O, 0.4 g/L MgSO_4_ 7-H_2_O, 0.72 g/L, K_2_H PO_4_, 0.72 g/L, KH_2_ PO_4_, and 0.01% (*w*/*v*) yeast extract. Bacterial growth was measured using a microplate reader and analyzed with Gen5^®^ software (version 2x). Triplicates of the test were performed [10].

### 2.7. Antimicrobial Activity

This activity was evaluated against *E. coli* ATCC25922 [19], *E. coli* O157:H7 [20], *Salmonella typhi* ATCC14028 [21], and *S. aureus* ATCC6538 [17] by diffusion plate assay, as reported by Reyes-Castillo et al. (2023) [10]. Absence of inhibition was considered as negative (−), weak inhibition as (+), and strong inhibition as (++). Antimicrobial resistance testing was conducted in triplicate. *L. casei* was used as a control [18]. Triplicates of the test were performed [9].

### 2.8. Antibiotic Resistance

This ability was assessed by the diffusion test, using Multibac discs for Gram-positive bacteria of Investigación Diagnostica, Ciudad de Mexico, Mexico, according to Reyes-Castillo et al. (2023) [10]. The discs contained 1 µg of dicloxacillin, 5 µg of ciprofloxacin, 10 µg of ampicillin and gentamicin, 15 µg of erythromycin, 25 µg of trimethoprim-sulfamethoxazole, 30 µg of cefotaxime, cephalothin, clindamycin, vancomycin, and tetracycline, and 10 U of penicillin. Triplicates of the test were performed. *L. casei* was used as a control.

### 2.9. pH Resistance

Prior to the experiment, a standard growth curve of *L. rhamnosus* and *L. casei* (control) was determined to establish the amount of CFU needed to inoculate 10 mL of MRS broth to obtain, after 8 h of incubation at 37 °C, 1 × 10^9^ CFU/mL. To prepare the inoculum of the desired concentration, 10 mL of MRS was inoculated and incubated at 37 °C for 24 h. Then, each culture was centrifuged for 5 min at 10,000× *g*, the pellet was washed twice with PBS (0.1 M, pH 7) and suspended in PBS adjusted to different pH values (1.5, 2, 3, and 5) using 1N HCl and incubated at 37 °C for 120 min. The viability was determined by the plate count method [18] at 0, 15, 30, 60, and 120 min. Using the following expression, the percentage of viability was determined under the test conditions. Triplicates of the test were performed [22].

$$\%\;Viability=\left(\frac{UFC/{mL}_{time\;min}}{UFC/{mL}_{time0\;min}}\right)*100$$

### 2.10. Hydrophobicity

Hydrophobicity was evaluated according to Vinderola et al. 2003 [23] to assess non-specific adhesion (the ability to adhere to hydrocarbons). Previously, a suspension of *L. rhamnosus* with an optical density (OD) of 1.0 at 600 nm (A0) was prepared. This suspension was vigorously mixed for 1 min with xylene in a 1:3 ratio and incubated at 37 °C for one hour. The aqueous component was separated, and its OD_600 nm_ was measured (A_1_). *L. casei* was the control strain. Triplicates of the test were performed. Hydrophobicity was defined as follows:

$$\%\;Hidrophobicity=\left(1-\left(\frac{A_{1}}{A_{0}}\right)\right)*100$$

### 2.11. Autoaggregation

Before the experiment, a bacterial culture was prepared as in the hydrophobicity test and was considered A_0_. The suspension was incubated at 37 °C for 2 h. Then, 0.1 mL was transferred and mixed with 1.9 mL of PBS. OD_600 nm_ was determined (A_1_). *L. casei* was the control strain. Triplicates of the test were performed [24]. Autoaggregation was defined as follows:

$$\%\;Autoaggregation=\left(1-\left(\frac{A_{1}}{A_{0}}\right)\right)*100$$

### 2.12. Coaggregation

This assay involved preparing a cell suspension as described in the hydrophobicity tests and mixing 1 mL of the suspension with an equal volume of each pathogen (*E. coli*, *S. typhi*, and *S. aureus*). The OD_600 nm_ was measured and designated as A_0_. The mix was then incubated for 2 h at 37 °C, after which the OD_600 nm_ was measured and considered as A_t_. *L. casei* was used as the control strain. Triplicates of the test were performed. The coaggregation percentage was defined as in Zuo et al. (2023) [24].

$$\%\;Coaggregation=\left(A_{0}-A_{T}/A_{0}\right)*100$$

### 2.13. Qualitative Assay of Bile Salt Tolerance

For bile salt tolerance assessment, the method of Gao et al. (2021) [9] was used. In brief, 10 μL of a fresh culture of the isolated strain was inoculated onto plates containing MRS agar containing 0.1%, 0.3%, and 0.5% *w/v* of glycocholic acid (GcCA), glycodeoxycholic acid (GDxCA), taurocholic acid (TcCA), taurodeoxycholic acid (TDxCA), and oxgall (OXGL) (Sigma Aldrich, St. Louis, MO, USA). Each assay was incubated aerobically for 48 h at 37 °C. The appearance of a halo surrounding the colony after incubation was considered an indicator of tolerance to bile salt (BS). Triplicates of the test were performed.

### 2.14. Bile Salt Hydrolase (BSH) Activity

This activity was assessed using whole cells (WC) according to González–Vázquez et al. (2015) [18], using an inoculum of 1 × 10^9^ CFU/mL in PBS containing 0.5% *w/v* of GcCA, GDxCA, TcCA, TDxCA, and OXGL and incubated for 48 h. *L. casei* was used as a control. A standard curve of glycine was established. Triplicates of the test were performed. BSH activity was calculated as follows:

$$\%\;BSH\;activity=\frac{{Glycin\;concentration}_{sample}}{{Glycin\;concentration}_{control}}*100$$

### 2.15. 2,2-Diphenyl-1 Picrylhydrazyl (DPPH) Radical Inhibitory Activity

To analyze the inhibition of DPPH radical activity by *L. rhamnosus* LBUX02, a solution containing 1 mL of 0.2 mmol/L of DPPH solubilized in CH_3_OH was mixed with 1 mL of WC or cell-free supernatant (CFs), or cell fragments Cfg, and maintained in darkness for 30 min. OD_517 nm_ was measured. *L. casei* was the control strain. Triplicates of the test were performed. DPPH inhibitory activity was calculated as described by Gao et al. (2021) [9]:

$$\%\;DPPH\;inhibitory\;activity=1-\frac{A_{sample}-A_{blank}}{A_{control}}*100$$

### 2.16. Hydroxyl Radical Inhibitory Activity

In this assay, 1 mL of WC, CFs, or Cfg was mixed with 1.0 mL of phenanthroline (2.5 mM), 1 mL of FeSO_4_ (2.5 mM), and 1 mL of PBS (pH 7.4), then the mixture was incubated at 37 °C for 90 min. To inhibit the reaction, 1 mL of 20 mM H_2_O_2_ was added, and the OD_517 nm_ was measured. *L. casei* was the control strain. Triplicates of the test were performed. Inhibitory ability was determined as described by Yan et al. (2019) [25]:

$$\%\;Hydroxyl\;radical\;inhibitory\;activity=1-\frac{A_{sample}-A_{blank}}{A_{control}-A_{blank}}*100$$

### 2.17. Superoxide Anion Radical Inhibitory Activity

The inhibitory activity was evaluated using 1 mL per sample of WC, CFs, or Cfg mixed with 3 mL of Tris–HCl solution (pH 8.2) and incubated at 25 °C for 20 min. Afterwards, 0.4 mL of pyrogallol (25 mM) was added, and the reaction was maintained at room temperature for 4 min. Then, 0.5 mL of HCl was added to inhibit the reaction, and OD_325_ nm was measured. *L. casei* was the control strain. Triplicates of the test were performed. Inhibitory activity was determined as follows [25]:

$$\%\;Superoxide\;anion\;radical\;inhibitory\;activity=(1-\frac{A_{sample}}{A_{blank}})*100$$

### 2.18. Qualitative Ferulic Acid Activity (EFA)

EFA activity on the plate was tested in agreement with the methodology reported by Tomaro-Duchesneau et al. 2012 [26]. Before the assay, LAB strains were inoculated in MRS broth supplemented with 1% ethyl 4-hydroxy-3-methoxycinnamate (EFA), 1.33 mM, and then they were incubated anaerobically under standard conditions. Meanwhile, MRS EFA agar plates were prepared using MRS agar (pH = 6.5 and 1.5% *w*/*v*). Once tempered, 0.3 mL of EFA (prepared at 10% *w*/*v* in N, N-dimethylformamide) was added per 20 mL of agar. Sterile Whatman #3 paper discs, impregnated with LAB strains (MRS-EFA culture), were placed onto the dried MRS EFA plates, and incubated at 37 °C for 48 h. No activity was considered as a negative (−) test, a moderate test was considered as positive (+), and total activity was considered as double positive (++). *L. casei* was the control strain. Triplicates of the test were performed.

### 2.19. Inhibition of α-Amylase

This experiment was carried out according to Won et al. (2021) [27]. In brief, a previous culture of *L. rhamnosus* was prepared. The experiment was carried out by plating the bacteria at a concentration of 1 × 10^9^ CFU/mL onto MRS broth and incubating at 37 °C for 24 h. Afterwards, the culture was centrifuged for 15 min at 8000× *g* at 4 °C. Then, 250 μL of α-amylase (pre-incubated at 25 °C for 10 min, 0.5 mg/mL) was mixed with 250 μL of CFs. The mix was then incubated at 25 °C for 10 min with 250 μL of 1% (*w*/*v*) starch solution in 0.02 M PBS. To finish the reaction, 500 μL of DNS dye reagent was added, and the test was boiled for 5 min, then cooled to room temperature and diluted fourfold with distilled water. OD_540 nm_ was determined using 1 mL of the diluted solution. *L. casei* was the control strain. Triplicates of the test were performed. The percentage of α-amylase inhibition was defined as follows:

$$\%\;\alpha -amylase\;inhibition=\left(\frac{{A_{control}-A}_{sample}}{A_{control}}\right)*100$$

### 2.20. Inhibition of α-Glucosidase

The inhibition of this enzyme was determined according to Won et al. (2021) [27]. *L. rhamnosus* strain (1 × 10^9^ CFU/mL) was grown on MRS broth under standard conditions. Then the culture was centrifuged at 8000× *g* for 15 min at 4 °C. A volume of 25 μL of supernatant was used to mix it with 150 μL of 0.01 M PBS (pH 7.0) and 75 μL of *p*-nitrophenyl glucopyranoside (PNPG) solution (0.2 M). This mixture was incubated at 37 °C for 10 min. To start the reaction, 50 μL of α-glucosidase (0.17 U/mL) was added. The reaction was incubated at 37 °C for 10 min, and it was finished by adding 1 mL of 0.1 M Na_2_CO_3_. OD_405 nm_ was quantified to determine the *p*-nitrophenol released. The experiment was performed in triplicate, with *L. casei* as the control strain. The percentage of α-glucosidase inhibition was defined as follows:

$$\%\;\alpha -glucosidase\;inhibition=\left(1-\frac{\left(C-D\right)}{\left(A-B\right)}\right)*100$$

### 2.21. Adhesion to Cancer Cell Lines

Cell adhesion assays were performed using different cancer cell lines, including SW-620 (colon cancer), Hella (cervical cancer), MCF-7 (breast cancer), HaCaT (aneuploid immortal keratinocyte cell), and SK-LU-1 (lung adenocarcinoma). Dulbecco’s Modified Eagle’s medium (Lonza, Basel, Suiza), with 5% fetal bovine serum (Gibco), 1% L-glutamine (Lonza, Basel, Suiza), and 1% antibiotics (penicillin/streptomycin, Gibco), was used for all cell line proliferations. Then, 4 × 10^5^ cells per well were seeded and incubated under standard conditions with 5% CO_2_. Triplicates of the test were performed [28].

Before the adhesion assay, a fresh culture of *L. rhamnosus* at 1 × 10^8^ CFU/mL was prepared. The bacteria were then incubated with the cancer cell lines at 37 °C with 5% CO_2_ for 1 h. The culture was washed with PBS and 300 µL of trypsin-EDTA (Gibco, Waltham, MA, USA). Adherent *Lactobacillus* cells were quantified by plate counting and expressed as CFU/mL. Adhesion was reported as the percentage of adhered bacterial cells with respect to the *Lactobacillus* initially added.

### 2.22. Statistical Analysis

Significant differences in all experiments were determined using the Kruskal–Wallis test, followed by Dunn’s post hoc test with a 95% confidence interval, performed using GraphPad Prism 5.01 software.

## 3. Results

### 3.1. Identification and Phylogeny

**Figure 1:**
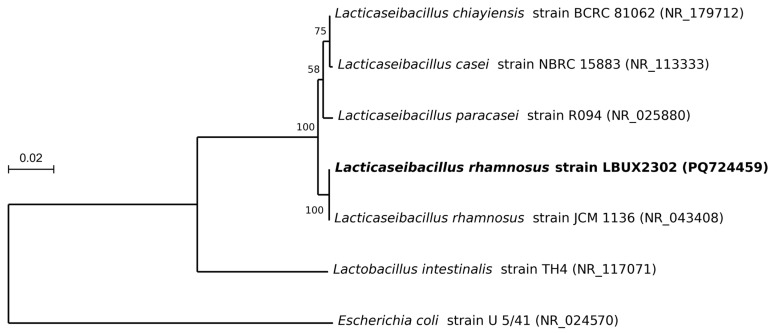
Phylogeny analysis based on 16S rRNA gene sequences from 7 bacteria including species of *Lacticaseibacillus, Lactobacillus* and *Escherichia*. The percent numbers at the nodes indicate the levels of bootstrap support based on Maximum Likelihood analyses of 1000 replicates.

The isolate obtained from stool samples exhibited a rod-like morphology and was Gram-positive. The DNA amplified via PCR was sequenced, and BLASTN (version 2.16.1+) analysis revealed 99.6% identity with *Lacticaseibacillus rhamnosus* (Figure 1). The strain was designated as *L. rhamnosus* LBUX2302 (GenBank accession: PQ724459.1).

### 3.2. Growth Kinetics on Different Carbon Sources

**Figure 2:**
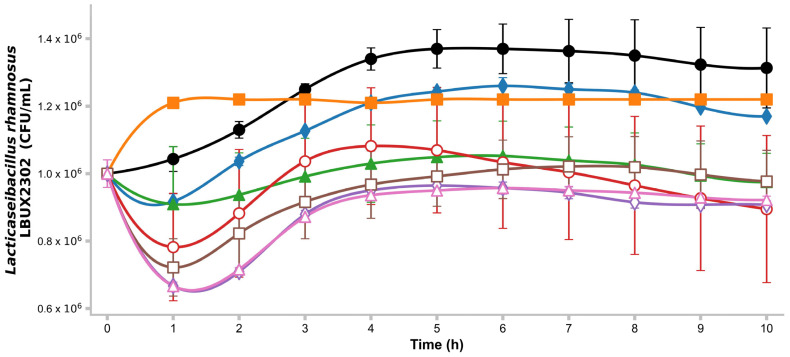
Growth kinetics of *L. rhamnosus* LBUX2302 by using different carbon sources. ● glucose, ♦ saccharose, ■ FOS, ▲ lactose, ○ raffinose, ◊ lactulose, □ xylan, and Δ xylose. Markers in each curve of growth kinetics represents the average of three independent trials. The error bars above the markers indicate the standard deviation.

*L. rhamnosus* LBUX2302 exhibited optimum growth at pH 7.0. and was able to metabolize all tested carbohydrates (Figure 2). The highest growth rate was observed in glucose (control). The exponential phase for glucose and saccharose lasted between 3 h, whereas for FOS, it was 1 h; however, no significant differences were detected. Differences (*p* ≤ 0.05) between glucose and lactose, raffinose, lactulose, xylan, and xylose were found. Sucrose showed significant differences compared to lactulose and xylose (Figure 1). Additionally, FOS exhibited significant differences compared to lactulose, xylan, and xylose. For raffinose, lactulose, xylan, and xylose, an initial phase was observed during the first hour, followed by an exponential phase between 1 and 3 h. After 4 h, the strain maintained stable metabolic activity over time (Figure 2).

Significant differences (*p* ≤ 0.05) in the growth of *L. rhamnosus* LBUX2302 when utilizing glucose vs. lactose, raffinose, lactulose, xylan, and xylose; sucrose vs. lactulose, and xylose; and FOS vs. lactulose, xylan, and xylose were found.

### 3.3. Catalase and Hemolysis

Neither *L. rhamnosus* LBUX2302 nor *L. casei* exhibited catalase or hemolytic activity. Conversely, *S. aureus* (negative control) displayed both activities, suggesting that the isolates are safe for use, which is an essential criterion for probiotic microorganisms (Table 1A).

### 3.4. Antimicrobial Activity

In the case of antimicrobial properties, *L. rhamnosus* LBUX2302 and *L. casei* strains inhibited the growth of pathogenic or non-pathogenic bacteria, either completely or moderately. The *L. rhamnosus* LBUX2302 strain totally inhibited *S. typhi* ATCC14028, a bacterium in which virulent genes such as *fmA* = fimbria production factor, *invA* = invasion factor, *SpvR* and *SpvC* = systemic infection (inhibition activation of macrophage), and *Stn* = enterotoxigenic substances production have been reported [21]. Also, it inhibited the *E. coli* ATTC25922 strain, a non-pathogenic strain commonly used as a control strain for antibiotic susceptibility testing [19]. In the case of *E. coli* 0157:H7, it was partially inhibited; this bacterium has shown pathogenicity that primarily affects children, the elderly, and immunosuppressed individuals, causing mild cases of diarrhea to hemolytic uremic syndrome [20]. *L. rhamnosus* LBUX2302 inhibited the *S. aureus* ATCC6538 strain, which has been used as a control for antibiotic susceptibility testing [17] (Table 1B).

### 3.5. Antibiotic Resistance

Probiotics must be evaluated for their antibiotic resistance and pathogenicity [17]. *L. rhamnosus* LBUX2302 and *L. casei* strains were sensitive to several antibiotics, exhibiting resistance to β-lactam and aminoglycoside antibiotics (Table 1C). *L. rhamnosus* LBUX2302 showed resistance to gentamicin (aminoglycoside), dicloxacillin, and penicillin (β-lactam antibiotics). *L. casei* specifically showed resistance to β-lactam antibiotics, such as vancomycin, dicloxacillin, and penicillin, and to protein synthesis inhibitors such as gentamicin.

### 3.6. Bile Salt Tolerance

Bile salt tolerance is an essential characteristic for evaluating the viability of LAB in the intestine. *L. rhamnosus* LBUX2302 and *L. casei* showed tolerance to primary and secondary bile salts at concentrations of 0.1%, 0.3%, and 0.5% (Table 1D).

### 3.7. Hydrophobicity and Autoaggregation

The cell surface hydrophobicity of *L. rhamnosus* LBUX2302 was 61%. The control strain did not exhibit hydrophobicity. A significant difference was observed between the strains. Autoaggregation in probiotics plays a fundamental role in their adhesion to the intestinal epithelium. *L. rhamnosus* LBUX2302 exhibited an autoaggregation rate of (53.5%), which was lower than that of the control strain (Table 1E).

### 3.8. Coaggregation

*L. rhamnosus* LBUX2302 showed a high average percentage of 70% of coaggregation with other microorganisms, specifically *E. coli* ATCC25922, *S. aureus* ATCC6538, and *S. typhi* ATCC14028, compared to the coaggregation ability of *L. casei* (Table 1F).

### 3.9. Ferulic Acid Activity

Both *L. rhamnosus* LBUX2302 and the control strain exhibited ferulic acid activity (Table 1G).

### 3.10. BSH Activity in CFs

**Figure 3:**
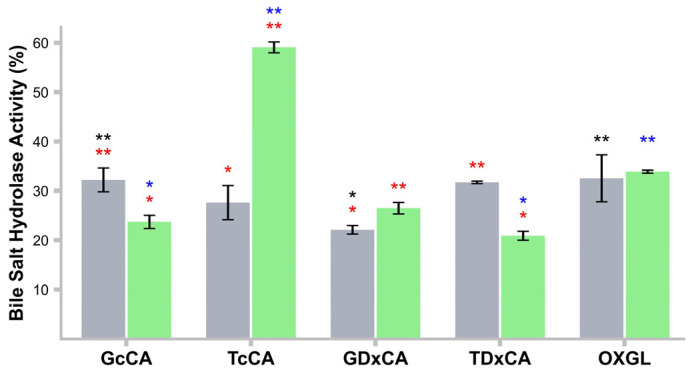
BSH activity of ■ *L. rhamnosus* LBUX2302 and ■ *L. casei*. * in black indicates significant differences *p* ≤ 0.05 between bile salts to *L. rhamnosus* LBUX2302, * in blue to *L. casei* and in red between both strains in the same bile salt. Each bar represents the average of three independent trials. Two bars that share the same number of asterisks of the same color indicate that there is no statistically significant differences between them. In contrast, bars with a different number of asterisks of the same color indicate a statistically significant difference. Error bars indicate the standard deviation.

Both *L. rhamnosus* LBUX2302 (32.2%) and *L. casei* (24%) exhibited BSH activity in GcCA, with no significant differences. Regarding TcCA, the control strain (58%) showed greater BSH activity than the *L. rhamnosus* LBUX2302 strain (30%). However, *L. rhamnosus* LBUX2302 (33%) displayed higher BSH activity in the presence of TDxCA versus the control strain (20%) (Figure 3). Significant differences in BSH activity were found in *L. rhamnosus* LBUX2302 between GcCA vs. GDxCA and GDxCA vs. OXGL. For *L. casei*, significant differences were found in GcCA vs. TcCA, TcCA vs. TDxCA, and GcCA and TDxCA with OXGL. When comparing both strains, significant differences were present in all bile salts except OXGL.

### 3.11. pH Survival

**Figure 4:**
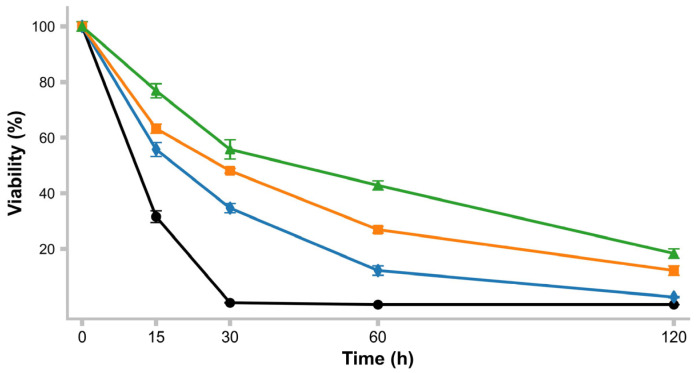
Percentage of survival under different pH 1.5 (●), 2 (♦), 3 (■), 5 (▲) of *L. rhamnosus* LBUX2302 and *L. casei*. Markers in each curve of viability curve represents the average of three independent trials. Error bars above the markers indicate the standard deviation.

The survival rate at different pH levels is shown in Figure 4. *L. rhamnosus* LBUX2302 was unable to survive at pH 1.5 for 120 min. However, at pH 2, 3, and 5, survival rates were 2.7%, 22%, and 27.5%, respectively, after 120 min, with no significant differences detected.

### 3.12. Antioxidant Activity

**Figure 5:**
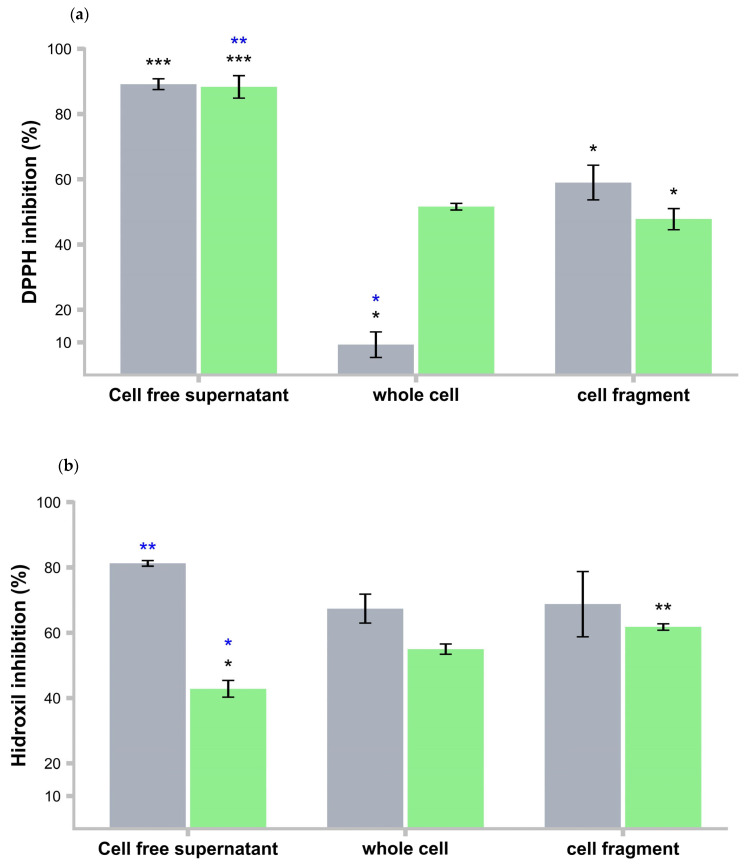

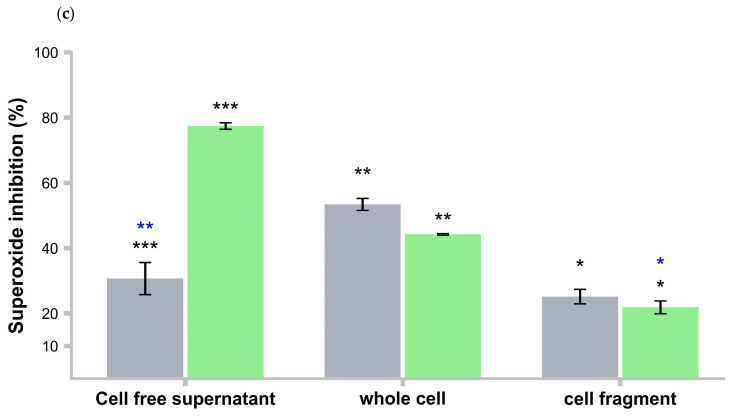
Antioxidant activity of ■ *L. rhamnosus* LBUX2302 and ■ *L. casei* (**a**) % of DPPH inhibition, (**b**) Hydroxyl anion inhibition, (**c**) Superoxide anion inhibition. Each bar represents the average of three independent trials. Error bars indicate the standard deviation. * in black means differences between different evaluations in the same strain. * in blue means differences among the strains between the treatments (*p* ≤ 0.05). Two bars that share the same number of asterisks of the same color indicate that there is no statistically significant differences between them. In contrast, bars with a different number of asterisks of the same color indicate a statistically significant difference.

The CFs of *L. rhamnosus* LBUX2302 and *L. casei* strains exhibited high antioxidant inhibition of DPPH. In contrast, the WC of *L. casei* demonstrated an antioxidant capacity 15 times greater than that of the *L. rhamnosus* LBUX2302 strain. For *L. rhamnosus* LBUX2302, a significant difference (*p* ≤ 0.05) was observed among the three treatments. Regarding *L. casei*, significant differences (*p* ≤ 0.05) were found between CFs and Cfg. (Figure 5a). The CFs of *L. rhamnosus* LBUX2302 were able to eliminate 80% of hydroxyl radicals, whereas the CFs of *L. casei* exhibited a 43% inhibition rate (*p* ≤ 0.05). No significant differences were observed among the different cell fractions of *L. rhamnosus* LBUX2302. However, for *L. casei*, significant differences (*p* ≤ 0.05) were found between CFs and Cfg. Additionally, significant differences (*p* ≤ 0.05) were observed between both strains in the CFs fractions (Figure 5b).

Regarding superoxide anion radical inhibition, *L. rhamnosus* LBUX2302 and the control stain exhibited significant differences (*p* ≤ 05) among their respective cell fractions. When comparing superoxide inhibition across the different cell fractions between the two strains, significant differences (*p* ≤ 05) were observed in both the CFs and the Cfg (Figure 5c).

### 3.13. α-Amylase and α-Glucosidase Activity Inhibition

**Figure 6:**
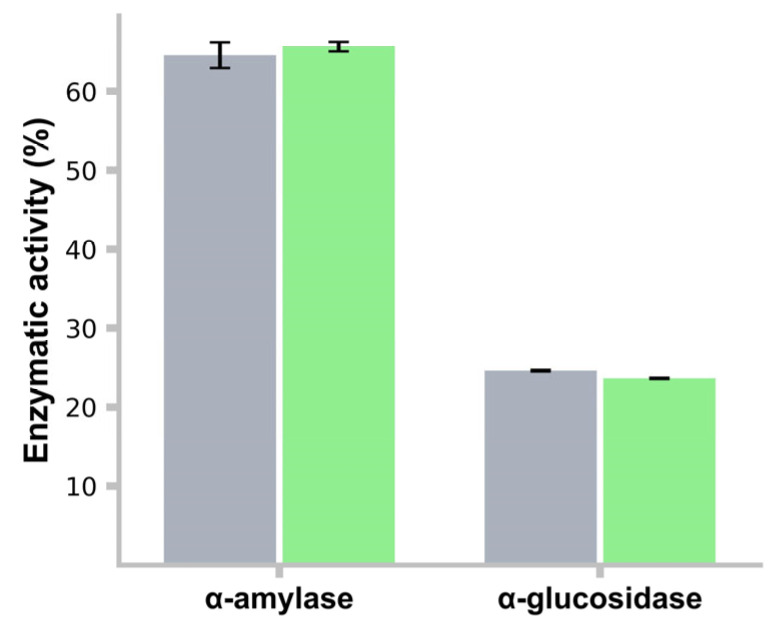
α-amylase and α-glucosidase activity inhibition of ■ *L. rhamnosus* LBUX2302 and ■ *L. casei*. Each bar represents the average of three independent trials. Error bars indicate the standard deviation. No significant differences were found.

The *L. rhamnosus* LBUX2302 strain exhibited 64.5% inhibition of α-amylase activity, showing no significant differences compared to the control strain (64%). Regarding α-glucosidase inhibition, *L. rhamnosus* LBUX2302 (23%) and the control strain (22.5%) demonstrated lower inhibition compared to α-amylase activity, with no significant differences (Figure 6). Between the two strains for either enzymatic activity, no significant differences were found.

### 3.14. Adhesion to Cancer Cell Lines

**Figure 7:**
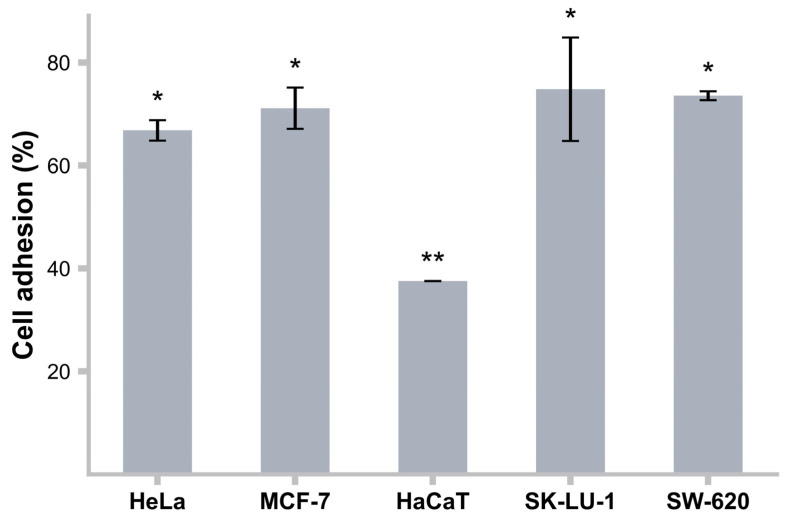
Cell adhesion ability of *L. rhamnosus* LBUX2302 to different cellular lines. * Indicates significant differences (*p* ≤ 0.05). Each bar represents the average of three independent experiments. Error bars indicate the standard deviation. Two bars that share the same number of asterisks indicate that there is no statistically significant differences between them. In contrast, bars with a different number of asterisks indicate a statistically significant difference.

*L. rhamnosus* LBUX2302 exhibited a higher adhesion capacity to MCF-7, SK-LU-1, and SW-620 cell lines (71%, 75%, and 74%) with respect to HaCaT (38%). These results suggest that adhesion depends on the origin of the cell line and its interaction with the strain species (Figure 7).

## 4. Discussion

The phenotypic characterization of probiotic microorganisms provides valuable information about their potential applications in the food, animal, and human health industries. The isolation of novel microorganisms with hypoglycemic and antioxidant properties requires systematic investigation. T2DM is a chronic disease characterized by hyperglycemia and insulin resistance. Genetic factors, dietary habits, obesity, sedentary lifestyle, and lack of exercise contribute to its development [29,30]. Postprandial glucose is considered the most important parameter in controlling the risk of diabetes. One of the strategies to keep postprandial glucose at adequate levels is the inhibition of the α-amylase and α-glucosidase enzymes, which catalyze the digestive process of carbohydrates. Conventional treatment involves pharmacological intervention, which may have adverse effects. An alternative approach is the use of probiotic bacteria to manage the disease [27,31,32]. The inhibitory activity shown by *L. rhamnosus* LBUX2302 (60% to 25%) over α-amylase and α-glucosidase is consistent with previously reported findings [31,33]. This inhibition may contribute to reducing blood glucose levels and slowing glucose absorption. Our results serve as an indicator for scaling up to in vivo evaluations to confirm the potential of *L. rhamnosus* LBUX2302 as a therapeutic agent for hyperglycemia. *Lactiplantibacillus plantarum* CCFM0236has demonstrated the ability to inhibit α-glucosidase and α-amylase, thereby reducing postprandial hyperglycemia. Huligere et al. (2023) [34] have demonstrated that different *Lacticaseibacillus* strains present in the human gut possess α-amylase and α-glucosidase inhibitory activity, reducing blood glucose responses in vitro.

*L. rhamnosus* LBUX2302 also exhibited strong antioxidant activity, particularly in the CFs, with high DPPH, hydroxyl, and superoxide anion inhibitory capacities. These antioxidative properties of *L. rhamnosus* LBUX2302 can be applied in fermented beverages and animal feed consumption since it is possible to reduce oxidative damage caused by stress and limit the use of synthetic antioxidants. This reduction in oxidative damage is probably due to the action of enzymes such as superoxide dismutase, glutathione peroxidase, glutathione reductase, and vitamins C and E, or to the action of bioactive peptides, which are polysaccharides such as the ones produced by the intestinal microbiota, as suggested by Lepecka et al. (2023) [35] Our results agree with those reported by Won et al. (2021) [27] and Wanget al. (2022) [36], who described *L. plantarum and L. paracasei* strains that showed high hypoglycemic, antioxidant, and probiotic properties. Oxidative stress, which implies a disequilibrium between the production of ROS and the defense system against oxidation, contributes to DM complications, the damage of genetic material, oxidation of proteins, and peroxidation of lipids [37]. Kaprasob et al. (2019) [38] have suggested that bioprocessing of cashews and apple juice through fermentation with *L. plantarum* and *L. casei* was effective for obtaining antioxidant nutraceuticals, which were relevant to DM2. Antioxidant activity has been reported for various LAB, including *L. rhamnosus*, *L. lactis*, and *L. plantarum* [39]. This activity has been associated with probiotic-induced glycemic improvement, suggesting that probiotics may modulate oxidative stress and contribute to glucose regulation [34,40].

Bile salt tolerance is essential for some probiotics to maintain viability in the intestinal environment. *L. rhamnosus* LBUX2302 and *L. casei* exhibited tolerance to various bile salt concentrations, which was mediated by the activation of surface proteins [41]. BSH activity plays a crucial role in the host metabolism, affecting energy regulation, lipid absorption, and cholesterol metabolism [42]. In this study, *L. rhamnosus* LBUX2302 and *L. casei* exhibited BSH activity in primary and secondary bile salts, as well as OXGL, which may contribute to cholesterol and triglycerides reduction [43]. Dong and Lee (2018) [44] have reported that BSH activity in *L. rhamnosus* E9 showed a preference for glycocholic bile salts. This activity could be attributed to the genes encoding this enzyme in *L. rhamnosus* [45].

On the other hand, *L. casei* exhibited a higher affinity for TcCA compared to other bile salts. Notably, in human bile salts, the proportion of glycine to taurine conjugates is 3:1 [46]. Similar BSH activity results have been reported for *L. casei* by various authors. González-Vázquez (2015) [18] identified BSH activity in *L. casei* Shirota using TcCA and TDxCA as substrates, with higher activity observed in TcCA. Lee et al. (2023) [47] have reported that supplementation with *L. reuteri* NCIMB 30242 could alleviate serum cholesterol levels by inhibiting intracellular cholesterol synthesis and promoting intestinal cholesterol excretion.

Autoaggregation, coaggregation, and hydrophobicity are key factors influencing bacterial adhesion [48]. *L. rhamnosus* LBUX2302 exhibited 54% of autoaggregation (Table 1E), indicating possible potential biofilm formation and prolonged colonization in the digestive tract [49,50]. A hydrophobicity assay revealed a 61% affinity to hydrocarbons (Table 1E), aligning with previously reported values for *L. rhamnosus* strains [51].

The autoaggregation of probiotic bacteria is an essential process for adhesion and is considered the first step in bacterial colonization [52]. This process leads to the formation of structured bacterial communities that facilitate cell-to-cell interaction and communication, the exchange of genetic material, adherence, and colonization in different environments [53]. Our results were superior to those reported by Grigoryan et al. (2018) [54] and Zawistowska-Rojek et al. (2022) [55], who have found autoaggregation percentages of 44.3% to *L. rhamnosus* INA-5.1%, 39.4% to *L. rhamnosus* R-2002, 10.1% to *L. rhamnosus* LrA, 14.6% to *L. rhamnosus* LrB, 15% to *L. rhamnosus* LrC, and 12.5% to *L. rhamnosus* LrD. In contrast, Sophatha et al. (2020) [56] have reported high autoaggregation levels in *L. rhamnosus* SD4 and SD11 (55–60%), which are similar to the results obtained in our study. The literature indicates that autoaggregation percentages can vary within the same species, likely due to differences in the probiotic strain’s origin and incubation time [57]. *L. rhamnosus* LBUX2302 showed a higher adhesion capacity in several cell lines, such as human colon cancer, cervical cancer, human breast cancer, and lung adenocarcinoma, which indicates that this microorganism could have new functional properties not yet explored. Thananimit, et al. (2022) [58] demonstrated that *L. rhamnosus* SD1, SD4, and SD11 exhibit high adhesion capacity in Caco-2 and HIEC-6 cells, with adhesion percentages ranging from 39% to 80%. However, no previous studies have reported the adhesion of *Lacticaseibacillus* in cell lines such as Hela, MCF-7, HaCaT, SK-LU-1, and SW-260. Evaluating adhesion in these cell lines could reveal new probiotic functions.

The coaggregation assay demonstrated strong adhesion of *L. rhamnosus* LBUX2302 to non-pathogenic and pathogenic bacteria, particularly *E. coli* ATCC25922, *S. aureus* ATCC6538, and *S. typhi* ATCC14028 (Table 1F), suggesting its potential to prevent pathogen colonization [59]. Our results were consistent with those reported by Cozzolino et al. (2020) [51], who found that *L. rhamnosus* GG exhibited high coaggregation with pathogenic bacteria, including *P. mirabilis* and *E. coli*. In contrast, Zawistowska-Rojek et al. (2022) [55] have reported lower coaggregation values for *L. rhamnosus* GG with *E. coli*, *S. typhimurium*, and *E. faecalis*. High coaggregation values indicate a strong capacity for barrier formation, which can prevent pathogenic colonization [59].

*L. rhamnosus* LBUX2302 showed weak antimicrobial inhibition against *E. coli* O157:H and *S. aureus* ATCC6538. Inturri et al. (2019) [60] have demonstrated that *L. rhamnosus* HN001 inhibited *E. coli* ATCC25922, *E. coli* ATCC35218, and *S. typhi* STN12. Davoodabadi et al. (2015) [61] have found that *L. rhamnosus* S19 inhibited various *E. coli* strains, including those with enteroaggregative, antihemorrhagic, antiinvasive, enteropathogenic, and enterotoxigenic activities. Furthermore, Johnson-Henry et al. (2008) [62] have reported that *L. rhamnosus* GG protects epithelial monolayers against the influence of *E. coli* O157:H7 on tight junctions claudin-1 and ZO-1.

The potential mechanism by which *L. rhamnosus* SCB0119 induces *S. aures* cell death may involve genomic instability due to interference with DNA repair pathways through the expression of the genes that encode ATP synthase, responsible for ATP hydrolysis [63]. Finally, probiotic antimicrobial activity can be mediated through multiple mechanisms, including organic acid production leading to acidification, bacteriocin production, competition for nutrients and adhesion sites in the host mucosa, and the induction of proinflammatory responses that facilitate pathogen elimination, both in vitro and in vivo [64,65,66]. Nonetheless, additional studies are required to elucidate the specific mechanism underlying the antimicrobial activity of *L. rhamnosus* LBUX2302.

The isolation of microorganisms with probiotic characteristics must include an evaluation of their abilities to tolerate relevant conditions, such as acidity and bile acids. The acidity that probiotics face in the gastrointestinal tract is a factor that influences their viability [67]. In this study, *L. rhamnosus* LBUX2302 exhibited a survival range between 22 and 27.5% at pH 2 and 3 over 120 min. Probiotics are intrinsically acid-resistant; they must withstand the harsh conditions of the stomach to reach the intestine alive, where they can colonize and exert beneficial effects [68]. The resistance of microorganisms to acidic pH may be attributed to a univariant gradient between cytoplasmic and extracellular pH, as well as proton extrusion via the F0F1-ATPase mechanism, which facilitates the survival in the gastrointestinal tract [69].

Safety is a crucial criterion for bacterial strains used for the food industry [70]. According to the European Food Safety Authority (EFSA), bacterial identification and hemolytic activity are among the primary criteria for assessing probiotic safety. In this study, we found no hemolytic activity in *L. rhamnosus* LBUX2302 or the control strain (Table 1A). Wang et al. (2021) [71] similarly reported an absence of hemolytic activity in LAB isolated from infant feces. Other LAB, such as *L. rhamnosus* CA15, *L. rhamnosus* CWKu-12, and *L. rhamnosus* SS73, have also been found to lack hemolytic activity [72,73] to produce hemolysin proteins, unlike the *S. aureus* species.

Antibiotics are one of the alternatives for treating infectious diseases caused by Gram-positive and Gram-negative bacteria in humans and animals. Their overuse has led to the emergence of antibiotic-resistant bacteria, which has caused an international public health problem [74]. Additionally, we assessed the antibiotic profile of *L. rhamnosus* LBUX2302, which exhibited resistance to gentamicin, dicloxacillin, and penicillin. LAB have been previously reported to exhibit intrinsic resistance to aminoglycosides (gentamicin) [75], which indicates that there are no genes that encode transferable resistance determinants and that it may be due to chromosomal zones of the bacteria [10]. Therefore, according to the qualified presumption of safety criteria, they should be considered safe. Furthermore, another author has suggested that the antibiotic resistance detected in *L. rhamnosus* GG is natural [76]. It is important to note that antibiotic-resistant LAB do not necessarily represent a health risk; nevertheless, additional research is required to confirm whether antibiotic-resistant genes in LAB are capable of being transferred [36]. In 2023, Shahali et al. [77] reported a high percentage of resistance to gentamicin, streptomycin, and ciprofloxacin in many LAB strains. The potential mechanism of gentamicin resistance in LAB strains is the absence of an antibiotic transporter, as gentamicin susceptibility is associated with the ability of the antibiotic to cross the bacterial membrane [78]. Previous studies have suggested that *Lactobacillus* spp. is susceptible to penicillin and β-lactams. Our findings indicate that *L. rhamnosus* LBUX2302 exhibited resistance to dicloxacillin and penicillin. These results are consistent with those reported by Hasan et al. (2020) [79], who found that *L. rhamnosus* MT539286 was resistant to the β-lactam antibiotics amoxicillin and oxacillin. Although *L. rhamnosus* is recognized as safe (GRAS) and has been granted a Qualified Presumption of Safety (QPS) status [80], it can still present antibiotic resistance and potentially transfer resistance genes to other bacteria via mobile genetic elements such as plasmids. Due to the need to perform a genomic characterization of *L. rhamnosus* LBUX2302, we are currently analyzing its genome to determine whether resistance to β-lactams and aminoglycosides is transferable or encoded at the chromosomal level. Specifically, we aim to identify intrinsic resistance genes related to the antibiotics gentamicin, penicillin, and dicloxacillin.

The *Lactobacillus* genus can efficiently grow on mono-, di-, tri-, and oligosaccharides [81]. Carbohydrates serve as an essential energy source for both the host and gut microbiota. In general, *Lactobacillus* species can metabolize these carbohydrates, producing compounds of interest for health and food industries [82,83]. In this study, we observed that *L. rhamnosus* LBUX2302 was able to metabolize monosaccharides and oligosaccharides (Figure 1), likely due to the expression of genes encoding glycosyl hydrolase enzymes. Our results are consistent with those reported by Ceapa et al. (2015) [84], who found that *L. rhamnosus* GG could utilize a wide range of mono-, di-, and polysaccharides, and polyols. In addition, Li et al. (2024) [85] have identified several genes encoding enzymes associated with carbohydrate metabolism in *L. rhamnosus* LR-ZB1107-01, including glycoside hydrolases, glycosyltransferases, esterases, and carbohydrate-binding modules. Furthermore, *L. rhamnosus* LR-ZB1107-01 possesses a complex phosphoenolpyruvate (PEP)–phosphotransferase system (PTS), which is responsible for the phosphorylation and transportation of sugars such as saccharose, lactose, maltose/glucose, mannitol, cellobiose, mannose, and fructose into the cell [85,86]. These sugar metabolism and transport systems are likely involved in the environmental adaptation capacity of *L. rhamnosus*. Moreover, we found that *L. rhamnosus* LBUX2302 can utilize FOS for growth, a result similar to the one reported by Kaewarsar et al. (2023) [87]. Additionally, Niu et al. (2023) [88] suggested that *L. rhamnosus* AS1.2466T can grow in the presence of FOS and, when administered simultaneously in mice, extends its colonization time in the intestine and ileum. The proposed mechanism for FOS degradation involves the fructanhydrolase enzyme, which has been identified in *L. paracasei* 1195 and functions in conjunction with the mannose PTS transporter complex [89]. However, this mechanism has not yet been identified in *L rhamnosus* species. Therefore, a key perspective for future research that we suggest is to sequence the complete genome of *L. rhamnosus* LBUX2302 and annotate the genes involved in all the activities determined in this study, and to test the effects in vivo models to demonstrate safety and corroborate the activities found.

## 5. Conclusions

This study indicates that *L. rhamnosus* LBUX2302 is a safe strain with promising probiotic properties. It demonstrates tolerance to bile salts, high hydrophobicity, and the ability to autoaggregate and congregate, along with strong adhesion capacity to MCF-7, SK-LU-1, and SW-620 cell lines. Furthermore, it exhibits significant hypoglycemic and antioxidant potential. However, further genomic characterization is necessary to confirm its safety and functionality at the genetic level. Once this characterization is completed, *L. rhamnosus* LBUX2302 could be considered for in vivo studies and potential applications in the pharmaceutical and food industries.

## Figures and Tables

**Table 1:** Biochemical and functional characterization of *L. rhamnosus* LBUX2302.

	**(A) Catalase**											**Hemolysis**										
*L. casei*	−											−										
*L. rhamnosus* LBUX2302	−											−										
*S. aureus*	+											+										
	**(B) Antimicrobial Activity**																					
	*E. coli* ATCC25922					*E. coli* O157:H7						*S. typhi* ATCC14028					*S. aureus* ATCC6538					
*L. casei*	+					+						+					+					
*L. rhamnosus* LBUX2302	++					+						++					+					
	**(C) Antibiotic Resistance**																					
	Va	Am	Stx		Ge			Dc		Cf		Clm		E		Pe	Te		Cfx		Cpf	
*L. casei*	r	s	s		r			r		s		s		s		r	s		s		s	
*L. rhamnosus* LBUX2302	s	s	s		r			r		s		s		s		r	s		s		s	
	**(D) Bile Salt Tolerance**																					
	GcCA			TcCA							GDxCA				TDxCA			OXGL				
	0.1	0.3	0.5	0.1			0.3		0.5		0.1	0.3	0.5		0.1	0.3	0.5	0.1		0.3		0.5
*L. casei*	r	r	r	r			r		r		r	r	r		r	r	r	r		r		r
*L. rhamnosus* LBUX2302	r	r	r	r			r		r		r	r	r		r	r	r	r		r		r
	**(E) % Hydrophobicity**											**%Autoaggregation**										
*L. casei*	0											94										
*L. rhamnosus* LBUX2302	61											54										
	**(F) % Coaggregation**																					
	*E. coli* ATCC25922								*S. aureus* ATCC6538							*S. typhi* ATCC14028						
*L. casei*	7.3								25							10						
*L. rhamnosus* LBUX2302	74								77							69						
	**(G) Ferulic Acid Activity**																					
*L. casei*	+																					
*L. rhamnosus* LBUX2302	+																					

(−) indicates absence of inhibition, (+) indicates weak inhibition, and (++) indicates strong pathogen and no pathogen inhibition. Va: vancomycin; Am: ampicillin; Dc: dicloxacillin; Cf: cephalothin; Pe: penicillin; Cfx: cefotaxime; Ge: gentamicin; Clm: clindamycin; E: erythromycin; Te: tetracycline; Cpf: ciprofloxacin, and Stx: trimethoprim-sulfamethoxazole. Sensitive (s); resistant (r) to antibiotics and bile salts. Glycocholic acid (GcCA), taurocholic acid (TcCA), glycodeoxycholic acid (GDxCA), taurodeoxycholic acid (TDxCA), oxgall (OXGL). Ferulic acid activity (+) indicates presence of activity or (−) indicates absence of activity.

## Data Availability

The genomic sequencing data of *L. rhamnosus* LBUX2302 have been registered in Gen Bank under the accession code PQ724459.1.

## Abbreviations

Am: ampicillin; BS: bile salt; BSH: Bile salt hydrolase; Cf: cephalothin; Cfg: cell fragments; CFs: cell-free supernatant; CFU: colony forming unit; Cfx: cefotaxime; Clm: clindamycin; Cpf: ciprofloxacin; Dc: dicloxacillin; DM2: type 2 diabetes; DNS: 3,5-Dinitrosalicylic acid; DM: Diabetes mellitus; DPPH: 2,2-diphenyl-1 picrylhydrazyl; E: erythromycin; EFA: ferulic acid activity; EFSA: European Food Safety Authority; FAO/WHO: Food and Agriculture Organization/codex alimentarius; FOS: fructooligosaccharides; GcCA: glycocholic acid; GDxCA: glycodeoxycholic acid; Ge: gentamicin; GRAS: generally recognized as safe; HaCat: spontaneously immortalized human keratinocyte cell line; HeLa: Henrietta Lacks cell line; H_2_O_2_: hydrogen peroxide; LAB: Lactic acid bacteria; NCBI: National Center of Biotechnology Information; MCF-7: breast cancer cell line; MRS: de Man Rogosa medium; OD: optical density; OH-: hydroxyl radicals; OXGL: oxgall; O^−2^: superoxide anion; PBS: Phosphate buffer solution; Pe: penicillin; PEP: phosphoenolpyruvate; PNPG: *p*-nitrophenyl glucopyranoside; QPS: Qualified Presumption of Safety; ROS: reactive oxygen species; SK-LU-1: human lung adenocarcinoma cell line; Stx: trimethoprim-sulfamethoxazole; SW620: adherent cell line isolated from the large intestine of a patient with Dukes-C colorectal cancer; TcCA: taurocholic acid; TDxCA: taurodeoxycholic acid; Te: tetracycline; Va: vancomycin; WC: whole cells.
